# Supplementary material for: Vaccine wastage rates and attributed factors in rural and urban areas in Uganda: Case of Mukono and Kalungu districts
Source: PLOS Glob Public Health. 2025 Jun 10;5(6):e0003745. doi: 10.1371/journal.pgph.0003745 (PMC12151339; doi:10.1371/journal.pgph.0003745)
Supplement: Attachment — Submitted filename: RESPONSE TO REVIEWERS _ VACCINE WASTAGE 2.docx [file pgph.0003745.s006.docx]

**Vaccine wastage rates and attributed factors in rural and urban areas in Uganda: Case of Mukono and Kalungu districts**

**Authors:** Mackline Ninsiima^1,2^*, Michael Muhoozi^1^, Henry Luzze^3^, Simon Kasasa^1^

**Institution affiliations**

^1^Department of Epidemiology and Biostatistics, School of Public Health, Makerere University, Uganda

^2^Uganda Public Health Fellowship Program, Uganda National Institute of Public Health, Kampala, Uganda

^3^ Ministry of Health, Kampala, Uganda

**Correspondence*:** Email: [nmackline@musph.ac.ug](mailto:nmackline@musph.ac.ug)

**POINT BY POINT RESPONSE TO REVIEWERS**

| **Comment** | **Response** |
| --- | --- |
| **Journal requirements** | |
| Please provide additional details regarding participant consent. In the ethics statement in the Methods and online submission information, please ensure that you have specified (1) whether consent was informed and (2) what type you obtained (for instance, written or verbal, and if verbal, how it was documented and witnessed). | We have specified that all eligible participants provided written informed consent, and all interviews were conducted on a voluntary basis. |
|  | |
| **Reviewer 1** | |
| **Abstract** | |
| The mention of the evaluation period for wastage should be moved up in the earlier part of the abstract and it should clearly state that wastage was tracked for 6 months as implied in the statement later presented that it was from March to August 2022. For the six-month period, was this prospective tracking of wastage or retrospective. This should be made clear. | Thank you for highlighting this concern. We have addressed it by specifying in the earlier part of the abstract that we estimated vaccine wastage rates for BCG, OPV, IPV, PCV, MR, and DTP-HepB-Hib over a six-month period, from March to August 2022, as per your guidance. |
| The abstract should also provide information about sample sizes for the study. Were all health facilities in the two districts included in the study? | The study was conducted in 22 health facilities, which were randomly selected from Mukono and Kalungu districts. We administered questionnaires to 57 health workers providing vaccination services and conducted 15 key informant interviews. |
| What type of vaccine wastage was monitored and reported, open and closed vial or just open vial? | We monitored closed and open vaccine wastage in health facilities. |
| The statement in lines 30 to 31 that “Vaccine wastage rates particularly for BCG and MR vaccines were attributed to compliance with Multi Dose Vial Policy (MDVP).” is not correct as all vaccines have guidance based on this MDVP. Rather, the higher wastage rates for these vaccines is because remaining doses in opened vials of these reconstituted vaccines without preservatives have to be discarded within 6 hours of reconstitution, as per the MDVP. The same MDVP states that remaining doses in opened vials of liquid vaccines with preservatives such as IPV, can be kept for 28 days. So the issue with the high wastage rate for MR and BCG is not the adherence to the policy, but due to the nature of the vaccines. | Thank you for this clarification. We have revised the statement based on your guidance. |
| **Introduction** | |
| In lines 79 to 81, the authors should not frame wastage as a bad thing always, given there is a tradeoff between wastage and coverage. As vaccinators try to minimize wastage, by implementing wastage reducing behaviors, such as not opening a vial for each eligible child, this also leads to missed vaccination opportunities. So the goal is not to minimize wastage as that may negatively impact coverage. I suspect national policy in Uganda is for vaccinators to open a vial even when one child is present and this increases wastage but the priority goal is increasing coverage. I would suggest that the statement you have on this on lines 85 to 87 be expanded to make it clear that wastage is not a bad thing, coverage is the priority and there is this tradeoff. | Revisions have been incorporated to reflect the need for the trade-off between wastage and coverage as follows:  Balancing the efficient provision of vaccines to targeted children with minimizing wastage from multi-dose vials is challenging because while reducing wastage is desirable, the primary goal is to ensure high vaccination coverage. Some level of wastage is acceptable and necessary to achieve broader public health objectives, as prioritizing coverage over wastage helps protect more children from preventable diseases. |
| The statement on lines 90 to 91 that reads “the inflexible rule surrounding the disposal of opened multi-dose vials at the end of a vaccination session or within a stringent six-hour timeframe….” is not correct. This is not just a stringent and unnecessary rule, this has to do with vaccine safety given the nature of the vaccines which do not have preservatives. | The statement has been revised as follows: While the aim is to optimize vaccine coverage, the safety requirement to dispose opened multi-dose vials at the end of a vaccination session or within six hours for vaccines without preservatives, unless the vaccine meets the criteria for use for up to 28 days after opening, leads to significant vaccine wastage rates. |
| The assessment done in 2014, as quoted in lines 94 to 95 is 10 years old and is outdated as things could have changed in the past 10 years. | Thanks for raising this concern. The statement has been deleted based on your guidance. |
| The reasons given in lines 119 to 123 seem to apply more directly to coverage rather than vaccine wastage. | Thank you for this insightful review. These reasons have indeed been deleted because they seem to apply more directly to coverage rather than vaccine wastage. |
| **Methods** | |
| Please clarify whether this was a prospective study using new tools or was the relying on routine immunization program data? | We relied on routine immunization program data as shown under the data collection section. Vaccine monitoring data for BGC, OPV, IPV, PCV, MR and DPT–HepB–Hib were extracted from Vaccine and Material Injection Control Book (VIMCB) and tally sheets using the abstraction tool from March–August 2022. |
| The study seems to have relied on immunization program data. If this is correct, did all the health facilities have reporting forms? What types of wastage were tracked on the forms. | Yes, all health facilities had the Vaccine and Material Injection Control Book (VIMCB) and tally sheets from which the data was extracted. However, vaccine wastage rates were not calculated prior so we had to calculate them based on these data variables: number of usable doses at the beginning of the month/starting monthly balance of vaccine doses, number of vaccine doses received during the month, number of usable doses in stock at the end of the month/monthly closing balance of doses, and number of doses administered/number of children vaccinated. |
| Lines 192 mentions a questionnaire, how when and how was the questionnaire completed and how frequently over the 6 month period? | The questionnaire was administered once at the beginning of the study by experienced research assistants among 57 health workers providing vaccination services in the selected health facilities to identify reasons for vaccine wastage. |
| **Results** | |
| Some of the tables and graphs don’t bring new insights and should be deleted. For example, Figure 1 duplicates a lot of the information in Table 1. The acceptable wastage rates could be added to Table 1 and this figure could be deleted. | Thank you for your recommendation. We have deleted Figure 2 and incorporated acceptable wastage rates into Table 1. |
| Also Figures 2 and 3 is not very informative as such trends are expected given variations in number of children vaccinated per year.  Averages of the months of the evaluation are more informative rather than month to month changes.  Given I don’t see the value of the trend analysis, Table 2 does not add value. | Thank you for your feedback. However, the authors agree that trend analysis is valuable because it reveals patterns and fluctuations in wastage rates over time that averages alone might miss. By examining month-to-month changes, we can identify specific periods of higher or lower wastage, which helps in understanding the underlying causes and planning targeted interventions. This dynamic view supports more informed decision-making and can guide efforts to improve vaccine management more effectively. |
| For Table 3, the results by rural urban are the same as in Table 1 given that all Kalungu is rural and all Mukono is urban and so the comparison of wastage rates for rural / urban is repeating what is stated already and should be deleted. | Thank you for your feedback. While it's true that all Kalungu districts are rural and all Mukono districts are urban, Table 3 provides a critical analysis of the significance of the difference in wastage rates between rural and urban areas. The p-values in Table 3 are essential for assessing whether the observed differences are statistically significant, which adds valuable context to our findings.  We believe that this additional layer of statistical analysis enhances the robustness of our results and provides a clearer understanding of the differences between rural and urban districts. |
| For Table 3, could you provide sample sizes for the subgroup based on ownership. Also provide ranges or some measure of dispersion as some of the averages need to be interpreted in the context of the small sample sizes. Similarly for Table 4, please provide sample sizes by facility type and some measures of dispersion. | Thank you for your valuable suggestions. Sample sizes for subgroups based on ownership and facility type have been provided in the Methodology section of the manuscript. We believe that these details, combined with the statistical analyses presented in Tables 3 and 4, offer a comprehensive understanding of the results. We agree with your point about the need for measures of dispersion to better interpret the averages, particularly given the small sample sizes. We have added ranges of vaccine wastage rates to Table 1 to enhance the clarity and robustness of the presented data. |
| How is the p-value being calculated in Table 4, what is the comparator? | For type of health facility, we applied Kruskal–Wallis H test to compare vaccine wastage rates since we had more than two categories of health facilities. This statistical test was selected to determine any differences in the average vaccine wastage rates because outcome data was not normally distributed. Significance was determined at a p–value of <0.05. |
| The reasons for vaccine wastage reported in the section starting on lines 314, is this based on observed wastage data or self-reported data from the health workers on what they know/think | The results referred to were obtained from administering questionnaires to health workers providing vaccination services in the selected health facilities, not from key informant interviews. This approach is detailed in the quantitative data collection section, where it is stated that a questionnaire, developed based on available literature, was administered among 57 health workers providing vaccination services in the selected health facilities to identify reasons for vaccine wastage. |
| The wastage results presented in the previous section do not disaggregate wastage rates by causes and if these data were collected, they should be reported. | Thank you for your feedback and for highlighting the importance of disaggregating wastage rates by causes. Unfortunately, we did not collect data on the specific causes of wastage in the current study. Our data collection focused on overall wastage rates without capturing the detailed breakdown by cause. We appreciate your suggestion and will consider it for future research to provide a more comprehensive analysis. |
| **Discussion** | |
| The word remarkable on lines 404 should be deleted. This finding is not remarkable as this has been reported elsewhere and is also not surprising. | The word “remarkable” has been deleted as per your guidance. |
| As mentioned before, the high wastage rate for BCG and MR is not due to the MDVP but rather to the vaccine presentation. Please correct lines 407 to 409. The same error is stated in line 427 and should be corrected as the application of the policy is because of vaccine safety considerations and should be adhered to! Again this incorrect information is stated on lines 435. | Thank you very much for your guidance. All highlighted statements have been revised to emphasize that the implementation of the policy is essential for ensuring vaccine safety. |
| Lines 410, how to errors and non-completion of wastage forms result in high wastage? | Errors and non-completion of vaccine monitoring tools have been deleted among the factors attributed to vaccine wastage. |
| The authors need to consider the tradeoff between wastage and coverage and note coverage is the higher priority. Their discussion on lines 436 to 439 need to be revised to make it clear that coverage is the priority. | We have revised the section to indicate that coverage should be prioritised over wastage. |
| I disagree with their discussion on lines 442 to 445 given the risk to missed vaccination opportunities, vials should be opened for each child present and enough vaccines should be availed for this. The policy recommendation should be to reduce the doses per vial.  Please see some recent articles on this including these two: Krudwig et al. The effects of switching from 10 to 5-dose vials of MR vaccine on vaccination coverage and wastage: A mixed-method study in Zambia. Vaccine 2020. DOI: 10.1016/j.vaccine.2020.07.012  Mvundura et al. Vaccine wastage in Ghana, Mozambique, and Pakistan: An assessment of wastage rates for four vaccines and the context, causes, drivers, and knowledge, attitudes and practices for vaccine wastage. Vaccine 2023. DOI: 10.1016/j.vaccine.2023.05.033  In line 453, single dose vials are not the solution as they have higher cold chain requirements. But rather vaccines with smaller doses per vial but still in multi dose vials. | On reviewing these articles, the authors concur with the suggestion to advocate for the manufacture of vaccines in reduced-volume multi-dose vials where feasible. |
| The discussion section is long and should be streamlined to focus on the key findings and the policy implications. For example, the authors discuss VVMs in lines 491 and yet their article did not mention this in any detail and so this is not part of their study. They did not characterize the availability of power at the study facilities or much else to warrant this discussion | Thank you for raising this concern. The authors request to maintain this paragraph because failure of the cold chain system characterized by VVM status was attributed to vaccine wastage, as per the qualitative results. |
| The allusions made in lines 509 about reconstitution are not related to wastage but rather to vaccine safety. Again, this information in these lines is incorrect as written and should be deleted.  The section on cost implications of vaccine wastage in lines 515 should also be deleted. The authors have not made such calculations in their analysis and so this should not be a discussion point. The discussion should be related to the results presented and not extrapolations from other publications.  Similarly, the discussion in lines 540 seems outside the scope of their findings and should be deleted. | The highlighted statements have been deleted based on your guidance. |
| **Conclusion** | |
| The wrong interpretations of the meaning of the MDVP is again stated in lines 573 and should be corrected. | This section has been revised with respect to your guidance. Thank you for the diligent manuscript review. |
| Some of the conclusions such as predictive modeling are not part of the study findings. This should be restricted to what is covered in the study findings. |  |
| As mentioned, single dose presentation is not the solution but rather lower vial containers. Single dose presentations present new challenges to the system and so this would be solving one problem and creating a new problem. |  |
| **References** | |
| The authors do not include some recent wastage studies in their review. Examples of the two studies cited above are excluded and some other recent studies on the topic. | The recommended references have been cited under the discussion section. |
| **Reviewer 2** | |
| In this study sponsored by the East African Regional Centre of Excellence, the authors have used quantitative and qualitative data to report vaccine wastage in Uganda highlighting several known challenges with suggestions for solutions taking the local context into consideration. While some wastage is acceptable understanding the factors that contribute to wastage is key to keeping wastage to acceptable rates. | Thank you for acknowledging the relevance of the study. |
| **Background** | |
| Some information on the schedule and vaccine vials used in country would be useful. | Thank you for raising this concern. We have added the vaccination schedule and vials utilized as follows:  The vaccination schedule aligns with WHO guidelines to ensure comprehensive disease coverage. Immunization starts at birth with BCG and OPV vaccines. At 6, 10, and 14 weeks, children receive OPV, IPV, Rotavirus, DTP-HepB-Hib, and PCV vaccines. At 9 and 18 months, they receive the Measles-Rubella vaccine. Most vaccines, including BCG, OPV, IPV, Rotavirus, DTP-HepB-Hib, and Measles-Rubella, are supplied in multi-dose vials, while PCV is administered using single-dose vials. |
| Which season was the data collected, rainy, dry? Give some indication | We collected data prospectively from March to August 2022, a period characterized by both the rainy season (March to May) and the transition to the dry season (June to August). This clarifies that the data collection period included both the rainy and dry seasons. |
| **Methods** | |
| Study design and Sampling procedures - Consider having subheadings for the quantitative and qualitative component. Consider the same for data collection and data analysis | Thank you for this recommendation. We have incorporated qualitative and quantitative sub-headings under the specified sections. |
| Besides one being rural and the other urban it is not clear how the two districts fit in the rest of the county. A map will help to show how these are placed in the rest of the country. Were these also chosen purposefully? | Mukono and Kalungu districts, both situated in Central Uganda, were selected based on their classification under Category 1, which denotes high vaccine access and utilization according to the 2019/20 WHO Reaching Every District (RED) Categorization for routine vaccination performance. They were purposefully chosen from the same category to minimize variations that could impact performance comparisons. |
| **Results** | |
| Line 314- 320 Reason for vaccine wastage Were these results from the interviews with the health workers? In that case, it would be better to move it to the section after the characteristics of the key informants. If not, please clarify in the methods how these data were collected. | The results referred to were obtained from administering questionnaires to health workers providing vaccination services in the selected health facilities, not from key informant interviews. This approach is detailed in the quantitative data collection section, where it is stated that a questionnaire, developed based on available literature, was administered among 57 health workers providing vaccination services in the selected health facilities to identify reasons for vaccine wastage. |
| Why do you having the footnote in table 4 with the p values, these can be seen in the table. | The footnote was included to clarify the interpretation of p-values, with asterisks denoting significance levels as follows: *p<0.05, **p<0.01, and ***p<0.001. |
| **Discussions** | |
| Line 453 – 455 - Though the challenge of cost was mentioned elsewhere in the manuscript and here consideration on feasibility of single dose vials is mentioned, perhaps a statement on the associated costs of single vial dose - increased storage space, higher vaccine cost could additionally be highlighted in the document. | Thank you for raising this concern. However, single-dose vials have been discouraged due to their higher cold chain requirements and increased storage needs. Instead, it is recommended to use lower-dose vials within multi-dose presentations. While single-dose vials might address one issue, they introduce new challenges to the system, making reduced-volume multi-dose vials a more practical solution. |
| **References** | |
| Review the references. There are some duplicates Reference 5 and 23, Reference 22 and 28 are the same Reference 7 is inappropriate for the statement in the text. | Thank you very much for your insightful review. We have revised the references based on your guidance. |
