# Supplementary material for: Vaccine wastage rates and attributed factors in rural and urban areas in Uganda: Case of Mukono and Kalungu districts
Source: PLOS Glob Public Health. 2025 Jun 10;5(6):e0003745. doi: 10.1371/journal.pgph.0003745 (PMC12151339; doi:10.1371/journal.pgph.0003745)
Supplement: Attachment — Submitted filename: RESPONSE TO REVIEWERS _ VACCINE WASTAGE.docx [file pgph.0003745.s007.docx]

**Vaccine wastage rates and attributed factors in rural and urban areas in Uganda: Case of Mukono and Kalungu districts**

**Authors:** Mackline Ninsiima^1,2^*, Michael Muhoozi^1^, Henry Luzze^3^, Simon Kasasa^1^

**Institution affiliations**

^1^Department of Epidemiology and Biostatistics, School of Public Health, Makerere University, Uganda

^2^Uganda Public Health Fellowship Program, Uganda National Institute of Public Health, Kampala, Uganda

^3^ Ministry of Health, Kampala, Uganda

**Correspondence*:** Email: [nmackline@musph.ac.ug](mailto:nmackline@musph.ac.ug)

**POINT BY POINT RESPONSE TO REVIEWERS**

| **Comment** | **Response** |
| --- | --- |
| **Editorial comments** | |
| 1. Thank you for uploading your study's underlying data set, (KALUNGU DISTRICT _ VACCINE WASTAGE DATA.xlsx and MUKONO DISTRICT _ VACCINE WASTAGE DATA.xlsx). We noticed that these files may contain potentially identifying participant information; specific location data and specific dates. Due to the inclusion of these potentially identifying data, we have removed these files from your file inventory. Please remove all personal information, ensure that the data shared are in accordance with participant consent, and re-upload a fully anonymized data set. Please note that spreadsheet columns with personal information must be removed and not hidden as all hidden columns will appear in the published file. | Columns with potentially identifying data have been removed from the datasets with reference to your guidance. Thank you… |
| **Reviewer 1** | |
| The methodology for the prospective data collection for vaccine wastage is still not clear. As we know, monitoring of vaccine usage / wastage is poor. So, can the authors better describe the process used by the study team during the six-month period to ensure that the health workers were monitoring and documenting vaccine usage? Was anything done to remind health workers to monitor and document vaccine usage? Or did the study team rely on routine data which that collected as usual (though we know monitoring of vaccine usage and wastage is poor due to several reasons such as lack of training, fear of high wastage rates as a negative performance indicator etc.). What was different at the study health facilities in the six-month study period versus what they routinely do? How can we know that the data over the six-month period reflect true vaccine usage and then wastage rates? | Thank you for these thoughtful comments. In response to concerns about the clarity of the methodology for vaccine wastage data collection, we revised the methods to include extra processes that were done to ensure accurate monitoring and documentation by health workers. These included initial training sessions, standardized data abstraction tools, periodic follow-up visits, and ongoing reminders from facility-level supervisors. Unlike routine practices, the study introduced reinforcement measures and cross-validation with stock records to address documentation gaps and ensure data reliability. Any inconsistencies were resolved through follow-up discussions, relying on standard tools like the Vaccine and Material Injection Control Book and tally sheets for accurate vaccine usage and wastage rates. Methods section Lines 154-161 and 210-225. |
| The response the authors provided in the response document to my question on whether the reasons for vaccine wastage presented in the results section are from observed wastage data or self-report should be explicitly stated in the revised manuscript. You mention in the response that “The results referred to were obtained from administering questionnaires to health workers providing vaccination services in the selected health facilities”. And so please state this in the revised manuscript otherwise readers can think these reasons are from the quantitative data on wastage rates and yet these are more the opinions of the health workers, which may or may not align with the quantitative data as this has not been validated. | We appreciate the reviewer’s feedback and agree that it is important to explicitly clarify the source of the reasons for vaccine wastage. In response, we have added the following statement before narrating the results lines 342-345.  *“The reasons for vaccine wastage presented in this section were obtained from self-reported responses collected through questionnaires administered to health workers providing vaccination services in the selected health facilities….”* |
| Please add to the limitations that you did not quantitative track wastage rate, as per your response to one of my prior questions and also to clarify the point above that the reasons for wastage are not from quantitative wastage data but self-report from health workers / vaccinators | Thank you for this important clarification, we have revised the limitations section to include the following statement in lines 561-567:  *This study did not quantitatively track individual vaccine wastage events at the point of administration but rather relied on aggregated facility reports extracted from vaccine monitoring tools. Additionally, the reasons for vaccine wastage presented in the results were self-reported responses by health workers through administered questionnaires rather than from direct quantitative analysis of vaccine wastage data since these can be observed in a trial setting. These self-reported reasons may not necessarily align with the recorded wastage rates, as no validation was conducted to compare the two sources of data.* |
| **Reviewer 2** | |
| Vaccine wastage rates and attributed factors in rural and urban areas in Uganda: Case of Mukono and Kalungu districts The authors have provided estimates of vaccine wastage from two districts in Uganda and used qualitative approach to provide factors associated with vaccine wastage. The factors reported are not new, however the paper highlights local challenges for these districts in Uganda. Here are some comments for consideration 1. In general there are several repetitions in the manuscript for example, Line 48/49 ‘in Uganda’ repeated, see ….line 168/69 for another repetition. They authors should review the manuscript and remove unnecessary duplication. | Thank you for this comment. We reviewed the manuscript and eliminated unnecessary duplications to ensure coherence in the manuscript. Lines 47, 50, 54, 86 and 98. |
| 2. A map showing the selected districts and or major health facilities would be helpful | A map has been added to illustrate health facilities where the study was done. |
| 3. Page 31 and 32. Some references are duplicated for example ref 5 and xxx. This looks like an error in the revised manuscript. Please review | References have been revisited and duplicates have been removed. |
| 4. The qualitative study confirmed low turnout for vaccination during the rainy season. Are six months within the rainy season in Uganda do they fall within the rainy season? This will affect the trends. Please include information on rainy season in relation to the months when the study was conducted. | Thank you for this comment. We appreciate the reviewer’s observation regarding the impact of the rainy season on vaccination trends. Uganda experiences two primary rainy seasons: March to May and September to November. The study period (March to August) includes one full rainy season (March–May) and a dry period (June–August) before the onset of the second rainy season. A statement to this effect has been added Methods section (data collection) lines 202-207. |
| 5. It appears that for resource constraints two districts were selected purposefully. However, it would be good to have some justification for the sample size. Why did you choose a third of the facilities in each district, was this based-on any proportion/size? How did you decide on the total numbers in the different categories to be chosen? | Thank you for this comment. We included the fact that first used proportionate to size sampling to ensure representation of all levels of facilities which yielded a pool where health facilities were eventually selected. Additional statements to make clear the justification for the selection has been added. Lines 183-184 and 190-192. |
| 6. Are Mobile vaccination clinics common in Uganda? | We acknowledge there are Mobile vaccination clinics in Uganda especially in rural areas and they can have effects on wastage. However, specific districts assessed did not report any mobile vaccination clinics during the assessed period. The study however, accounts for wastages during static and outreaches which is the commonest set-up countrywide. |
| 7. Line 417, what is the point of referring to recommendation by Min of Health in India? | Thank you for this observation. Comparison made to India whose context is very different was not required. We have deleted to only maintain similar studies in similar contexts. |
| 8. Line 435/6 give more context to the comparison of IPC and OPV vaccine vial presentation in the setting you are referring to. | Thank you for this observation. We included additional context on the comparison of IPV and OPV vaccine vial presentation in our setting, highlighting the differences in utilization, administration methods, and resulting wastage rates. Lines 472-479. |
| 9. There are several statements without supporting references/in appropriate ref Line 70-72 provide references Line 75 ref 6 is inappropriate Line 119-125 provide some references Line 436-38 provide some reference | Thank you for this observation, relevant references have been included. |
